# A Refined and Efficient CNN Algorithm for Remote Sensing Object Detection

**DOI:** 10.3390/s24227166

**Published:** 2024-11-08

**Authors:** Bingqi Liu, Peijun Mo, Shengzhe Wang, Yuyong Cui, Zhongjian Wu

**Affiliations:** 1 Norla Institute of Technical Physics, Chengdu 610041, China; liubingqi@cdu.edu.cn (B.L.);; 2 School of Mechanical Engineering, Chengdu University, Chengdu 610106, China

**Keywords:** object detection, remote sensing images, deep learning, RE-YOLO

## Abstract

Remote sensing object detection (RSOD) plays a crucial role in resource utilization, geological disaster risk assessment and urban planning. Deep learning-based object-detection algorithms have proven effective in remote sensing image studies. However, accurate detection of objects with small size, dense distribution and complex object arrangement remains a significant challenge in the remote sensing field. To address this, a refined and efficient object-detection algorithm (RE-YOLO) has been proposed in this paper for remote sensing images. Initially, a refined and efficient module (REM) was designed to balance computational complexity and feature-extraction capabilities, which serves as a key component of the RE_CSP block. RE_CSP block efficiently extracts multi-scale information, overcoming challenges posed by complex backgrounds. Moreover, the spatial extracted attention module (SEAM) has been proposed in the bottleneck of backbone to promote representative feature learning and enhance the semantic information capture. In addition, a three-branch path aggregation network (TBPAN) has been constructed as the neck network, which facilitates comprehensive fusion of shallow positional information and deep semantic information across different channels, enabling the network with a robust ability to capture contextual information. Extensive experiments conducted on two large-scale remote sensing datasets, DOTA-v1.0 and SCERL, demonstrate that the proposed RE-YOLO outperforms state-of-the-art other object-detection approaches and exhibits a significant improvement in generalization ability.

## 1. Introduction

In recent years, the rapid development of the aviation industry and the advancement of precision sensor technology have led to exponential growth in various types of remote sensing data. Precise positioning and accurate identification of objects in remote sensing images (RSIs) are of great significance in many fields, such as resource utilization, geological disaster risk assessment, urban planning and management [1,2,3,4,5]. However, for RSIs, traditional methods that rely on manual labeling or annotation are time-consuming, labor-intensive and subject to human bias, making them unsuitable for the rapid response needed in emergency situations. In addition, RSIs are captured from a top-down perspective, which presents unique challenges not encountered in natural scene images. These challenges include the diverse scales and dense distributions of objects, where small targets are often obscured by complex background noise [6,7]. Therefore, there is a growing demand for automatic and accurate detection of targets in RSIs, drawing considerable attention of numerous researchers from both industry and academia.

Traditional object-detection methods typically rely on manually designed features and classifiers, such as sliding window-based approaches represented by HOG [8] and DPM [9], region-extraction methods exemplified by selective search [10] and edge box [11], and segment-based techniques like GrabCut [12]. These methods extract features from candidate regions within an image and classify them using support vector machines (SVMs). However, the reliance on manual selection and parameter adjustment severely limits the flexibility and adaptability of these approaches, leading to suboptimal performance and poor generalization capabilities. With the rapid advancement of deep learning, convolutional neural networks (CNNs) have become increasingly prevalent in object-detection tasks in RSIs. The mainstream deep learning-based object-detection algorithms can be broadly categorized into two types: two-stage detectors and one-stage detectors. Two-stage detectors first generate region proposals from the input image and then classify these regions. A seminal example is the Region-Based Convolutional Neural Network (R-CNN) [13], which was the first to integrate deep learning technology into the field of image object detection. To enhance the efficiency and performance of R-CNN, Fast R-CNN [14] and Faster R-CNN [15] were subsequently developed, achieving superior performance compared to traditional machine learning methods. Despite their excellent accuracy, these two-stage detectors are often constrained by high computational complexity and slow inference speeds, making them less suitable for scenarios requiring real-time performance. To overcome the limitations of two-stage detectors, one-stage detection algorithms have emerged, achieving a balance between detection accuracy and computational efficiency. One-stage detectors regard detection as a regression problem of a single network, with typical examples including single shot multibox detector (SSD) [16], you only look once (YOLO) [17,18,19,20,21,22,23] and RetinaNet [24]. These algorithms significantly reduce inference time by predicting both bounding boxes and class probabilities in a single network. However, one-stage detectors tend to be less sensitive to smaller-scale objects, which can limit their applicability in certain contexts. To mitigate the limitations of one-stage detectors, particularly in detecting small objects, the feature pyramid network (FPN) [25] was introduced. FPN utilizes a pyramid-like architecture to extract and fuse multi-scale features from different levels of feature maps, improving the accuracy of object detection to a certain extent. Building on the FPN framework, numerous improvements and derivative algorithms [26,27,28] have been proposed, further enhancing the detection performance of objects at various scales.

Although the aforementioned methods have achieved promising results in object detection for natural images, remote sensing images (RSIs) present unique challenges. These challenges include low detection accuracy for small targets and difficulty in detecting densely arranged objects. To tackle these challenges, a refined and efficient object-detection algorithm (RE-YOLO) is designed based on the YOLO pipeline. The key contributions are outlined as follows:

(1) The refined and efficient module (REM) and RE_CSP block are proposed, enabling effective multi-scale feature extraction with minimal computational cost. Furthermore, RENet, constructed by stacking RE_CSP blocks, serves as the backbone network, offering strong feature-extraction capabilities. Experimental results demonstrate that the proposed method outperforms other state-of-the-art methods in detection performance.
(2) To facilitate the fusion of multi-scale hierarchical and spatial features, a spatial extracted attention module (SEAM) is designed to establish long-range dependencies. It can be effectively combined with the RE_CSP block to generate attention maps that promote representative feature learning and capture richer semantic information, further improving the model’s performance in detecting small targets.
(3) Shallow feature extraction and multi-scale feature-fusion strategy are crucial for RSIs, determining whether the network can accurately identify densely arranged targets of varying scales in complex backgrounds. This paper proposes a three-branch path aggregation network (TBPAN), which aims to enhance the positional and salient information extracted from low-level feature maps. TBAPN incorporates additional branches between layers at different levels to establish cross-scale connections, enabling an effective multi-scale fusion of shallow features with deep semantic information. Experimental results demonstrate that TBPAN significantly alleviates the problem of missed detection for dense small targets and greatly improves the detection performance.

## 2. Preliminary

This section outlines the significant concepts and definitions related to remote sensing object detection as discussed in this paper. Table 1 presents the key abbreviations and symbols employed throughout the paper, along with their full names and explanations.

This paper is organized as follows: Section 2 illustrates the important concepts and definitions as discussed in this paper. Section 3 reviews the related works relevant to our method. Section 4 provides a detailed introduction to the proposed RE-YOLO algorithm. Section 5 describes the datasets used in the experiments and discusses the experimental results. Finally, Section 6 gives a brief discussion and conclusion of the paper.

## 3. Related Works

### 3.1. Remote Sensing Object-Detection Framework

In recent years, object detection has received extensive attention in the field of computer vision. Most existing object detectors are designed with the assumption that the target objects are aligned along the horizontal axis, making them primarily suitable for detecting objects in natural images. These detectors are broadly categorized into two groups: two-stage detectors represented by the Faster RCNN family [13,14,15] and one-stage detectors represented by SSD [16] and YOLO [17,18,19,20,21,22,23]. Two-stage detectors generally offer higher accuracy but come with increased model and computational complexity, while one-stage detectors are typically simpler in structure, more efficient and better suited for industrial applications. However, Compared with natural images, RSIs present unique challenges, such as higher resolution, complex backgrounds, large variations in object scale, numerous small objects and dense object arrangements. These factors contribute to missed detections and make the detection task more challenging. To address these issues, specialized algorithms have been developed for object detection in RSIs. For instance, SCRDet [7] enhances the representation ability of the network by designing a sampling fusion network tailored for small object detection. StrMCsDet [6] improves the recognition accuracy of targets in RSIs by generating a single-stage feature mapping architecture within a cross-stage partial network. CF2PN [29] boosts the fusion of multi-scale features by incorporating a cross-scale fusion module (CSFM). LP-YOLO [3] achieves fast and effective recognition of landslides in remote sensing images by building a lightweight feature-extraction backbone. Additionally, LSKNet [30] incorporates large convolution kernels within the network to better handle contextual variations of objects in remote sensing scenes, thereby enhancing the recognition accuracy.

In recent years, achieving a better trade-off between performance and efficiency has become a key research direction in image processing tasks. Lightweight network architectures like MobileNetV1-V3 [31,32,33], ShuffleNetV1-V2 [34,35], GhostNet [36,37] and FasterNet [38] have emerged as feature-extraction backbones, aiming to achieve fewer parameters and enhanced network performance. For instance, MobileNet employes depthwise convolution (DWC) and pointwise convolution (PWC) to approximate the functionality of standard convolutional layers, achieving comparable performance while significantly reducing computational costs. ShuffleNet utilizes the group convolution (GC) and shuffle operation to facilitate information flow among various groups. GhostNet minimizes redundant feature maps by applying linear transformations to only half of the spatial features. Additionally, RepVGG [39] introduces a re-parameterization strategy that converts multi-branch structures in the training phase into a single-path architecture for inference, reducing computational complexity and memory usage while maintaining high accuracy. VoVNet [40,41] introduces One-Shot Aggregation (OSA), which aggregates features from multiple layers at once, reducing complexity and parameters while enhancing the network’s ability to capture diverse information. CSPNet [42] introduces a feature reorganization strategy that divides the feature map into two parts and merges them at different layers, reducing parameters and computational complexity while improving the network’s ability to capture diverse information. Inspired by these works, the one-stage detector framework based on YOLO is adopted in this study. The lightweight REM and RE_CSP blocks are designed to achieve enhanced feature representation and fewer parameters. Specifically, the REM integrates meticulously designed GC, PWC and a pooling layer, ensuring efficient feature extraction and reduced redundancy. Furthermore, RENet is proposed as an efficient backbone by stacking RE_CSP blocks, demonstrating that an optimized network architecture can achieve a better trade-off between performance and efficiency in remote sensing image-detection tasks.

### 3.2. Multi-Scale Feature-Fusion Strategy

One of the primary challenges in object detection is to effectively represent and process multi-scale features in images. Early detectors typically relied on the pyramid feature hierarchy directly extracted from the backbone network to make predictions. The feature pyramid network (FPN) [25] proposed an innovative top-to-down pathway that aggregates multi-scale features, enabling cross-scale connections and information exchange across different feature layers produced by the backbone network, thereby greatly enhancing the representation ability of the output features. However, the unidirectional information flow in FPN limits the effectiveness of information fusion. To address this limitation and utilize contextual information more effectively at different scales, the Path Aggregation Network (PANet) [26] was developed, adding an additional bottom-up pathway to the FPN framework to facilitate more comprehensive information fusion. Recently, numerous improved algorithms based on FPN have been proposed to enhance multi-scale feature representation and fusion, yielding notable results. For example, Sun et al. [43] proposed a bidirectional feature-fusion module (Bi-DFFM) for SAR ship detection, fully exploiting cross-scale features. M2det [44] introduced a U-shaped module to enhance multi-scale feature fusion, while ABNet [45] designed an adaptive feature pyramid network (AFPN) that adaptively fuses multi-scale features across different channels and spatial locations. EfficientDet [27] introduced a novel bidirectional repeatable module (BiFPN) to improve the efficiency of information fusion across different levels. In more recent efforts, DAMO-YOLO [46] has adopted Reparameterized Generalized-FPN (RepGFPN) to enhance the information flow between the backbone and neck. EAL-YOLO [47] proposed Attentional scale Sequence Fusion P2-Neck (ASF2-Neck) to enhance the model’s ability to detect small target defects. Similarly, Gold-YOLO [48] introduced an advanced Gather and Distribution (GD) mechanism that leverages convolution and self-attention to strengthen multi-scale feature-fusion capabilities.

While these methods have shown excellent performance and have significantly alleviated the problem of information loss between feature layers of different scales, most are not tailored for object detection in RSIs. Due to the high resolution of RSIs, two critical issues are often overlooked. The first is the extraction of shallow texture information and its effective interaction with deep semantic features. The second is the inefficient cross-layer information exchange and the resulting information loss, which ultimately constrain detection performance in RSIs. To address these challenges, a three-branch path aggregation network (TBPAN) is proposed. The TBPAN can generate high-quality feature presentations for each scale by effectively fusing fine-grained features from the adjacent lower level. Additionally, TBPAN adds an extra branch to enhance the contribution of shallow texture information in the final fusion layer, thereby improving overall model performance.

### 3.3. Semantic Information Exploitation

Mining semantic information is crucial for understanding the relationship between complex environments and detection targets. Extensive research indicates that accurate detection often requires rich contextual information [30,49,50,51], while limited features can hinder correct classification. Therefore, a deep exploration of semantic information is particularly critical for RSIs characterized by complex backgrounds. To enhance the network’s representation capability, a series of attention modules are integrated into the network to facilitate better semantic information extraction. The SE block [52] compresses the feature map into a feature vector via global average pooling, learns the importance weights of the channels through two fully connected layers and subsequently applies these weights to the feature map to highlight significant features. Similarly, attention modules such as DANet [53] and CBAM [54] simultaneously model spatial and channel attention, adaptively learning the importance of features across different channels and spatial dimensions, thereby bolstering the network’s ability to represent contextual information. In addition, Hou et al. [55] introduced the Coordinate Attention (CA) mechanism [56] to enhance the YOLOX model’s capacity for information extraction and integration, addressing the challenges of landslide detection in RSIs. Chen et al. [57] proposed spatial and channel attention modules, SCA_C and SCA_T, which operate on Convolutional and Transformer layers respectively, enabling dual fusion of spatial and channel features across multiple scales and further enhancing model performance. Lv et al. [58] incorporated the CA block into the neck to capture more comprehensive contextual semantic information, promoting farm aerial imagery scene recognition accuracy. In this study, the spatial extracted attention module (SEAM) is designed to tackle the challenges of small target recognition in RSIs. Specifically, the SEAM is incorporated into the bottleneck of the backbone network ($P_{4}$ and $P_{5}$ layers) to promote robust feature learning and enrich semantic features, ultimately improving the recognition performance for small targets.

## 4. Main Results

In this section, the overall architecture of the proposed RE-YOLO is presented. Following this, a detailed description of its key components is provided, including the REM, RE_CSP block, RENet, the design of the SEAM and the structure of TBPAN.

### 4.1. Proposed Methods

**Figure 1:**
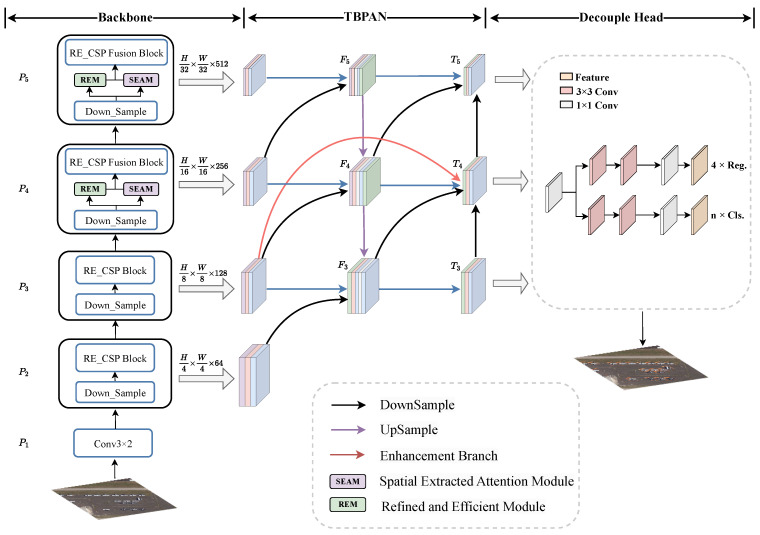
Architecture of our proposed RE-YOLO.

The overall structure of the proposed RE-YOLO is shown in Figure 1. RE-YOLO follows the one-stage detector paradigm and consists of three primary parts: the feature-extraction backbone network RENet, the three-branch path aggregation network (TBPAN) with the spatial pyramid pooling feature (SPPF) layer [59] and the decouple detection head. The RENet is composed of four key components, namely REM, RE_CSP block, SEAM and RE_CSP Fusion block. Unlike the traditional CSPDarkNet, RENet is stacked by a series of lightweight RE_CSP blocks, which significantly reduces the number of parameters and computational overhead. Each RE_CSP block incorporates the REM, which is designed with a 3 × 3 group convolution block, a 1 × 1 pointwise convolution block and a pooling layer. Furthermore, RENet integrates the Spatial Extracted Attention Module (SEAM) to enhance the extraction of edge and semantic information from RSIs.

As illustrated in Figure 1, the detection process begins with the input remote sensing image, denoted as $Input\in R^{H\times W\times 3}$, which is fed into the backbone network. The backbone network extracts hierarchical features through a series of processing steps, involving two RE_CSP blocks, two RE_CSP Fusion blocks and the corresponding down-sampling layers. This process produces feature maps at levels 1–5, denoted as $P_{i}$, where i∈{1,2,3,4,5}. Each $P_{i}$ has dimensions $\in R^{\left(H/2^{i}\right)\times \left(W/2^{i}\right)\times 2^{i}C}$, where C = 16. The previous two RE_CSP blocks produce low-level layer $P_{2}$ and $P_{3}$, which retain more detailed texture and positional features. The following two RE_CSP Fusion blocks produce high-level layers $P_{4}$ and $P_{5}$, which preserve richer semantic information. The RE_CSP fusion block is generated by the integration of the RE_CSP block with the SEAM. To better capture contextual information, SEAM is utilized to establish long-range dependencies and is fused with RE_CSP block, thereby enhancing the relevance of low-level features. Specifically, the output of layer $P_{3}$ is split into the two branches after the down-sampling layer. One branch is sent directly to RE_CSP block to extract image features, while the other branch is processed by SEAM to generate a weighted attention map. The outputs from these branches are then fused to produce the final output. Following five stages of feature extraction, the tensor $P_{5}\in R^{(H/32)\times (W/32)\times 512}$ is obtained and subsequently fed into the neck network, TBPAN. TBPAN incorporates an additional P2 layer and branches at different levels to achieve enhanced fusion of shallow information with deep semantic features. The resulting fused features, denoted as $T_{i}$, where i∈{3,4,5}, are then fed into networks for category classification and bounding box regression. Similar to [23], our detection head employs a decoupled structure, separating the classification and regression tasks into two independent network branches. Prior studies [23,60,61] have demonstrated that such decoupled heads can significantly improve both performance and convergence speed.

### 4.2. REM and RE_CSP Block

The backbone network of the detectors is typically composed of substantial 3 × 3 conventional and 1 × 1 convolution blocks, such as ResNet50 and CSPDarkNet53. These networks rely heavily on traditional convolutional blocks, resulting in numerous parameters and significant computational demands. In recent years, with the development of various lightweight networks, modules such as depthwise convolution (DWC), group convolution (GC) and pointwise convolution (PWC) have been widely used in these networks [31,35,36,41,62], serving as key components to alleviate computational burden. According to [34], to increase the number of channels without significantly raising the FLOPs, two methods are employed: PWC and channel split structure. Furthermore, CSPNet [42] employs a forking strategy on input feature maps to reduce parameters and computation while enhancing the network’s capacity for multi-scale feature extraction. Specifically, the output feature maps usually contain significant redundancy and many feature maps are highly similar [36]. Inspired by these works, the REM and RE_CSP block are designed as a feature-extraction module to minimize this redundancy while effectively capturing multi-scale information.

**Figure 2:**
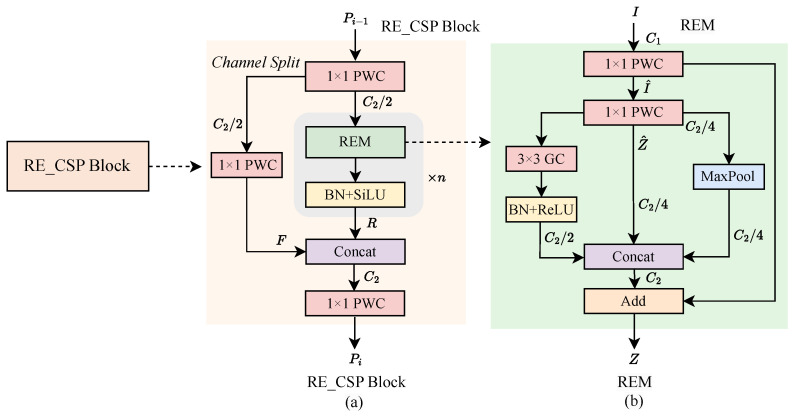
The structure of the proposed REM and RE_CSP Block. $C_{1}$: The number of input channels. $C_{2}$: The number of output channels. PWC: pointwise convolution. GC: group convolution.

As illustrated in Figure 2b, the REM consists of four components, two 1×1 PWC layers, a 3×3 GC block and a pooling layer. The $C_{1}$ and $C_{2}$ denote the number of input and output channels, respectively. We carefully balanced the $C_{2}$ ratio across the three modules, assigning the output channels of the GC, PWC, and pooling layer to $C_{2}/4$, $C_{2}/2$ and $C_{2}/4$, respectively, followed by concatenation. This configuration facilitates multi-scale feature extraction while effectively reducing redundancy. The process of REM is as follows: Initially, given an input $I\in R^{H\times W\times C_{1}}$, *I* is processed by the first PWC layer to integrate information across the channel dimension, producing the richer semantic representation, denoted as $\hat{I}$. To mitigate the redundancy in feature maps, $\hat{I}$ is further passed through the following PWC layer with the channels reduced to $C_{2}/4$, resulting in a refined feature map $\hat{Z}$, $\hat{Z}\in R^{H\times W\times 0.25C_{2}}$. Next, $\hat{Z}$ is processed through two separate paths: one using a max-pooling layer with $C_{2}/4$ kernels to capture prominent spatial features and another using a 3×3 GC block with $C_{2}/2$ kernels to enhance multi-scale feature extraction. This dual-path processing enhances the network’s multi-scale capabilities while reducing computational complexity. Subsequently, the outputs of these operations are concatenated, the transformed feature map $\hat{Z}$ and $Z\in R^{H\times W\times C_{2}}$ are described in Equations (1) and (2):

(1) $$\hat{Z}=PWC_{2}\left(PWC_{1}\left(I\right)\right),$$

(2) $$Z=Concat(\left[\hat{Z},ReLU\left(BN\left[GC\left(\hat{Z}\right)\right]\right),Pooling\left(\hat{Z}\right)\right])+I,$$

where *I* represent input feature maps, $PWC_{i}$ denotes pointwise convolution, BN refers to batch normalization [63], Concat denotes concatenation operation, Pooling refers to max-pooling layer and GC refers to group convolution with the kernel size of 3.

Our feature-extraction backbone network, RENet, is composed of multiple RE_CSP blocks, which are designed to extract global semantic and multi-scale information across various depths. As shown in Figure 2a, the REM is a critical component of the RE_CSP block of residual connections, where the “n” represents the number of REMs used in different depths within RE_CSP block. RE_CSP block is inspired by CSPNet, comprising three PWC layers and REM. During the feature-extraction process of RE_CSP block, the input feature maps, expressed as $P\in R^{H\times W\times C}$, undergo a channel-split operation, where the channels are divided into two halves and sent to two parallel branches. In the first branch, *P* is directly passed through the left PWC layer to generate the transformed feature map $F\in R^{H\times W\times C/2}$. In the second branch, *P* is processed through a dense block consisting of the PWC layer and the REM, producing the outputs $R\in R^{H\times W\times C/2}$. The feature maps *F* and *R* are then concatenated along the channel dimensions and the final PWC layer is applied to integrate features. The process of final output feature map $P_{l}$ at layer *l* can be described in Equations (3)–(5):

(3) $$F=PWC_{1}\left(P_{l-1}\right),$$

(4) $$R=SiLU(BN\left[REM\left(PWC_{2}\left(P_{l-1}\right)\right)\right]),\;l\in (2,3,4).$$

(5) $$P_{l}=PWC_{3}\left(Concat\left[F,R\right]\right),$$

where $P_{l-1}$ denotes the input feature map *P* at layer (l−1), BN refers to batch normalization and Concat denotes concatenation operation.

### 4.3. Spatial Extracted Attention Module

The ability to mine semantic information and integrate contextual details is particularly crucial for detecting small objects in complex environments. In the feature-extraction phase of the backbone network, high-level feature maps typically contain richer semantic features but retain less texture and positional information. The expansion of the receptive field further contributes to the loss of local details, degrading small object-detection performance. Recent studies [57,64] suggest that incorporating a specific attention mechanism can adaptively learn the importance of features across different channels and spatial dimensions, thereby mining deeper semantic information and enhancing the network’s ability to represent contextual information. In this study, a lightweight Spatial Extracted Attention Module (SEAM) is designed to address the challenge of accurately recognizing small objects in complex environments. The SEAM can utilize stacked cross-convolutional blocks to establish long-range dependencies along both vertical and horizontal dual-channel directions, generating attention maps. It is integrated into the backbone at the $P_{4}$ and $P_{5}$ layers, promoting representative feature learning. Specifically, the attention maps generated by SEAM further enhance the acquired semantic features through weighted fusion with the REM in the RE_CSP Block, improving the network’s recognition performance.

**Figure 3:**
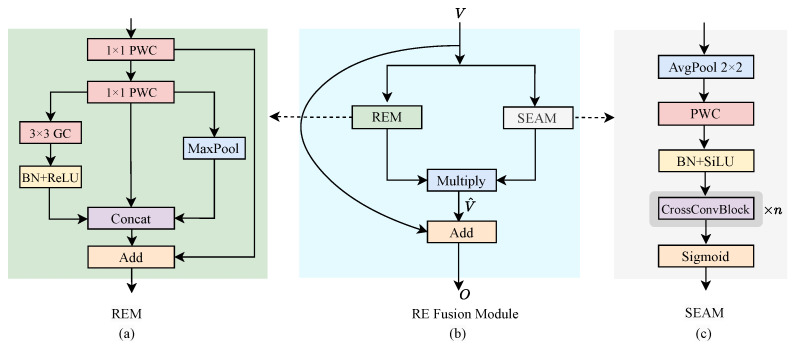
The architecture of SEAM and fusion details.

As shown in Figure 3, the architecture of SEAM and fusion details are illustrated. In the feature-fusion process, the input feature $V\in R^{H\times W\times C}$ is processed in parallel by the REM and SEAM branches. In the REM branch, the output feature is denoted as REM(V). In the SEAM branch, *V* first passed through an average pooling operation to squeeze its height and width by a factor of 2, followed by a PWC layer for spatial compression, producing $P\in R^{H/2\times W/2\times C}$. The following Equation (6) represents this process:

(6) P=PWC(AvgPool(V)).

Next, CrossConvBlocks are applied to enhance the diversity and richness of the features. A Sigmoid operation generates an attention map that assigns weights from 0 to 1 across spatial locations, capturing the importance of different regions. The resulting attention map, denoted as SEAM(V), is described in Equation (7):

(7) $$SEAM\left(V\right)=Sigmoid\left(CrossConv(SiLU(BN[P]))\right),$$

where *V* denotes the input feature map, CrossConv represents CrossConvBlock layer, BN refers to batch normalization and SiLU [65] represents the activation function.

Finally, the obtained feature REM(V) and the attention feature map SEAM(V) are combined using element-wise multiplication to produce the fused feature map *O*. The fusion process is expressed in Equation (8):

(8) O=REM(V)⊙SEAM(V)+V,

where *V* denotes the input, REM(V) represents the output after the REM layer with input V, SEAM(V) denotes the output after the SEAM layer with input *V*, ⊙ denotes element-wise multiplication operation and *O* represents the fused feature map.

### 4.4. Structure of TBPAN

The purpose of multi-scale feature fusion is to aggregate features at different resolutions to effectively identify objects of various scales. Generally, feature layers at different levels carry information about objects of varying sizes. The low-level feature layers retain finer edge and texture details crucial for detecting small objects, whereas the higher-level feature layers provide high-dimensional representations that capture richer semantic information, which is essential for recognizing larger objects.

Multi-scale features extracted by the backbone network can be denoted as $P^{in}\in \left\{P_{l1}^{in},P_{l2}^{in},P_{l3}^{in},\ldots \right\}$, where $P_{l1}^{in}$ represents the features at level l1. The purpose of the multi-scale feature-fusion strategy is to find a way to effectively aggregate features of different scales and feed them into the decoder for further accurate object detection, which involves both recognition and localization. Traditional top-down FPN [25] is inherently limited by its unidirectional information flow, which can restrict the efficacy of feature fusion. To address this limitation, PANet [26] adds an additional bottom-up path aggregation network to better merge features. Furthermore, BiFPN [27] proposes bidirectional cross-scale connections at the same level to achieve more comprehensive fusion within each layer. Although these methods have shown excellent performance in natural image detection, they are not entirely optimized for detection in RSIs. For remote sensing applications, effectively extracting shallow texture features and developing robust multi-scale feature-fusion strategies are critical for detection, which determines whether the network can accurately identify and locate densely arranged objects and targets of varying scales in complex environments. However, the aforementioned methods mentioned above often overlook the extraction of shallow texture features.

**Figure 4:**
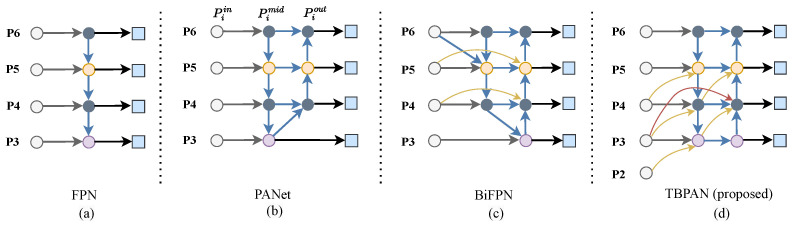
The architecture of TBPAN in comparison with FPN, PANet, and BiFPN.

As shown in Figure 4, these methods typically begin by obtaining input features $P^{in}\in \left\{P_{3}^{in},P_{4}^{in},P_{5}^{in},\ldots \right\}$ starting from P3, where $P_{i}^{in}$ represents a feature map level with resolution of 1/$2^{i}$ of the input images. Additionally, both PANet and BiFPN lack direct interaction between feature nodes. This means that the features of intermediate nodes are often derived from multiple convolutions and sampling operations applied to either upper or lower levels, leading to minimal contributions from these levels to the feature network. Consequently, there are issues with the low efficiency of cross-layer information exchange and significant information loss. To enhance shallow information extraction and improve the efficiency of multi-scale feature fusion, a novel strategy tailored for RSIs, called the three-branch path aggregation network (TBPAN), is proposed. As shown in Figure 4, from left to right, the nodes in the columns are designated as $P_{i}^{in}$, $P_{i}^{mid}$, $P_{i}^{out}$, i∈(2,3,4,5,6). $P_{i}^{in}$, $P_{i}^{mid}$, $P_{i}^{out}$ represent the feature maps at the input node for backbone, intermediate node and output node for prediction, respectively. Firstly, TBPAN introduces the feature $P_{2}^{in}$ into $P_{3}^{mid}$ to strengthen the contribution of bottom-level features in the fusion network. Secondly, to avoid the loss of information during the fusion of features between intermediate and upper nodes, TBPAN merges these features directly through a single down-sampling operation. As illustrated in Figure 4d, unlike the bidirectional cross-scale connections of the same level proposed by BiFPN, TBPAN incorporates cross-scale connections between different levels (gold line) to ensure comprehensive integration of shallow texture information and deep semantic features. Thirdly, TBPAN adds an extra branch (red line) to enhance the contribution of shallow texture information in the final fusion layer, thereby promoting the network’s capacity to capture contextual information. Equation (9) represents the process for $P_{4}^{out}$ feature:

(9) $$P_{4}^{out}=P_{4}^{mid}+Cross\left(P_{3}^{in}\right)+Down\left(P_{3}^{in}\right),$$

where Cross (·) denotes the cross-scale connection operation of different-level points, and Down (·) represents the down-sampling operation. Experiments have shown that TBPAN significantly alleviates the problem of missed detection of dense small targets and greatly enhances the performance of target detection in RSIs.

## 5. Experiment Results

### 5.1. Dataset

To evaluate the performance of the proposed method in object-detection tasks for remote sensing images, the large-scale remote sensing dataset DOTA-v1.0 [66] was utilized. Additionally, to verify the effectiveness and generalization capability of the model on high-resolution remote sensing images, the Southwest China Earthquake Region Landslide (SCERL) dataset was established with higher resolution for evaluation.

(1) DOTA-v1.0 Dataset: This dataset consists of remote sensing images captured by various sensors and platforms, containing 2806 high-resolution aerial images of different sizes. Given that the dataset includes numerous small-sized targets, similar to the approach in [30,67], each image was cropped into sub-images of size 1024×1024 with an overlap of 200 pixels to ensure smoother detection tasks. For the experiments, attention was focused on the five categories with the most instances for training and evaluation: small vehicle, large vehicle, ship, plane and storage tank. The training set comprises 6253 images, while the validation set contains 1794 images, all resized to 1024×1024 pixels.
(2) SCERL Dataset: This dataset primarily consists of remote sensing landslide images from the Longmenshan area of Sichuan Province. It includes 5434 images in the training set and 1461 images in the validation set with resolution of 2000×2000, which were cropped to 640×640. The dataset features a wide range of object sizes and includes a single category for Landslide. The SCERL dataset poses several challenges due to varying imaging conditions, such as differences in weather, lighting and overall image quality, making it a comprehensive test for evaluating object-detection performance in complex environments.

### 5.2. Evaluation Metrics

To evaluate the detection performance of the model on RSIs, a series of evaluation indicators are employed in the experiments. Similar to evaluation metrics used in many papers [56,57,68,69], these metrics are divided into two categories. The first category evaluates the accuracy of the model, including Intersection over Union (IoU), Precision (P), Recall (R), F1 score, Average Precision (AP) and mean Average Precision (mAP). The second category refers to the evaluation of the model network size, including the number of model parameters (Params), single-image inference speed and floating-point calculation (FLOPs). IoU [68] is used to evaluate the overlap between the predicted box and the ground truth (GT) box. IoU is defined as in Equation (10):

(10) $$IoU=\frac{\mathrm{area}(B_{p}\cap B_{gt})}{\mathrm{area}(B_{p}\cup B_{gt})},$$

where $B_{gt}$ denotes the GT box, $B_{p}$ denotes the predicted box. Precision and Recall [69] are fundamental metrics used to evaluate the quality of detection models. Precision represents the proportion of data that are true positive samples among all samples predicted as positive by the model, while Recall indicates the proportion of actual positive samples correctly identified by the model. They are defined as follows in Equations (11) and (12):

(11) $$Precision=\frac{TP}{TP+FP},$$

(12) $$Recall=\frac{TP}{TP+FN},$$

where TP (True Positive) represents the number of correct predictions made by the model, FP (False Positive) represents the number of incorrect positive predictions and FN (False Negative) represents the number of missed detections.

The F1score is a crucial evaluation metric, especially for classification and detection tasks, as it strikes a balance between Precision and Recall. F1score is defined in Equation (13):

(13) $$F1\;score=2\times \frac{Precision\times Recall}{Precision+Recall}.$$

The Average Precision (AP) serves as a comprehensive metric that simultaneously considers both the Precision (P) and Recall (R) of a model, making it a crucial evaluation criterion for object-detection tasks. AP is computed as the area under the Precision–Recall (P-R) curve [68], and is defined in Equation (14):   

(14) $$AP=\int_{0}^{1}P\left(R\right)d\left(R\right).$$

A$P_{50}$ and A$P_{50:95}$ are evaluation criteria that assess the model’s performance at different Intersection over Union (IoU) thresholds, providing a more nuanced understanding of its localization accuracy. Specifically, A$P_{50}$ represents the average precision at an IoU threshold of 0.5, while A$P_{50:95}$ considers the mean average precision computed across multiple IoU thresholds, ranging from 0.5 to 0.95 in increments of 0.05. A$P_{50:95}$ is widely regarded as a more comprehensive and rigorous metric, as it better reflects the model’s overall detection performance under varying degrees of overlap. Mean Average Precision (mAP) aggregates the AP scores across all object categories to evaluate the model’s overall performance, and is described in Equation (15):

(15) $$mAP=\frac{1}{N}\sum_{n=1}^{N}AP_{i}^{n},$$

where *N* represents the number of categories and *i* represents the IoU threshold.

### 5.3. Training Setting

All experiments in this study were constructed using the open-source tool Ultralytics [23] for training and evaluating models on the NVIDIA Tesla P100-16GB GPU. The development environment used for all experiments and model implementation was PyCharm 2021.2.2 Professional Edition. During the experiments, the training data was augmented using random flips with a probability of 0.5, mosaic augmentation and HSV saturation enhancement. The number of training epochs was set to 150, with the optimizer performed using Stochastic Gradient Descent (SGD) with a momentum of 0.937, and an initial learning rate of 0.01. For the DOTA-v1.0 dataset, multi-scale data (640 and 1024) were utilized for training and validation, while the input image size for the SCERL dataset was standardized to 640. Considering the memory constraints, the batch size of a single GPU was set to 24 for an input image size of 640×640 and reduced to 10 for an input image size of 1024×1024. The loss functions employed were consistent with the baseline YOLOv8, including cross-entropy loss for classification, CIoU loss and DFL loss for bounding box localization. In addition, the regression utilized an anchor-free approach. The training process of our proposed RE-YOLO is represented in Algorithm 1.
**Algorithm 1:** Pseudocode of Training Process of RE-YOLO
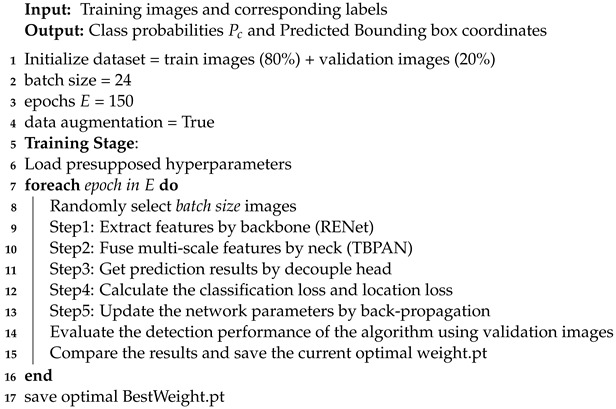


### 5.4. Ablation Studies

In this section, the effectiveness of the designed backbone RENet, SEAM and TBPAN is verified through ablation experiments conducted on the DOTA-v1.0 dataset. Comparisons are made with modules of similar functions in existing advanced methods. Specifically, CSPDarkNet, which serves as the feature-extraction backbone network in YOLOv8, corresponds to RENet in this study. PAFPN, the feature-fusion network of PANet and YOLOv8, corresponds to the proposed TBPAN. SE, the attention module introduced in SENet, corresponds to the proposed SEAM.

Aiming to gain a clearer understanding of the model’s performance under different input sizes and to objectively verify the adaptability and robustness of the proposed modules to scale transformation, a detailed analysis was conducted on the performance benefits when training with input image sizes of 640 and 1024. In addition to measuring the metric values, the number of parameters (Params) and the floating-point computations (FLOPs) for the networks were also calculated under the same experimental environment. The ablation experiment results, presented in Table 2, demonstrate that the modules integrated into RE-YOLO can effectively enhance the detection performance of the network. As shown in Table 2, models trained with larger input sizes consistently outperform those with smaller input sizes in terms of detection metrics. For the baseline model YOLOv8s, increasing the input size to 1024 leads to improvements in Recall, mA$P_{50}$ and mA$P_{50:95}$ by 2.8%, 1.7% and 1.3%, respectively, compared to an input size of 640. Similarly, the proposed RE-YOLO shows improvements of 1.9% in Recall, 1.5% in mA$P_{50}$ and 2.4% in mA$P_{50:95}$, along with an additional increase of 0.6% in Precision. These results suggest that a larger input size provides more pixel-level information and finer details, enabling the model to learn more accurate target features. RENet, serving as the backbone network of the RE-YOLO, aims to balance accuracy and computational complexity. Under the same network structure, substituting CSPDarkNet with RENet as the backbone results in slight improvements in Precision and mA$P_{50:95}$, while achieving a reduction in the number of parameters by approximately 18.0% (2.0 MB) and a decrease in FLOPs by 21.5% (6.2 G).

**Figure 5:**
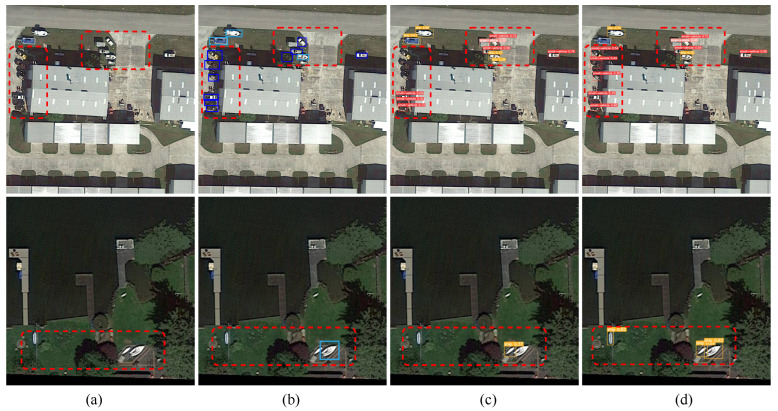
Comparison of detection results before and after incorporating TBPAN in the RE-YOLO framework. (**a**) Source image. (**b**) Ground truth. (**c**) RE-YOLO with PAFPN. (**d**) RE-YOLO with TBPAN.

The TBPAN is designed to enhance the extraction of shallow information and improve multi-scale feature fusion, effectively addressing the problem of missed detection of dense small targets in RSIs. Compared with BiFPN [27], TBPAN achieves a 1.4% improvement in Precision, a 0.6% increase in Recall, a 0.4% enhancement in mA$P_{50}$ and a 1.0% increase in mA$P_{50:95}$ when the model is trained with an input size of 640. It is worth noting that the use of the BiFPN greatly reduces the overall number of parameters of the network. Compared with PAFPN [23], TBPAN improves Precision by 1.6%, Recall by 0.5%, F1 score by 1.0%, mA$P_{50}$ by 0.6% and mA$P_{50:95}$ by 1.8% on a model with an input size of 640. However, this performance gain comes with an increase in Params and FLOPs, attributed to TBPAN’s capacity to extract more low-level feature channels during feature fusion, thereby adding to the computational complexity. Figure 5 visualizes the detection results of RE-YOLO using TBPAN and PAFPN as neck-fusion networks, with red boxes highlighting areas of significant improvement. As shown in the first row, the “small vehicle” category located in a shadowed environment is not well detected by PAFPN (Figure 5c), particularly the small vehicle on the left side of the image. This illustrates the limitations of PAFPN in extracting shallow texture features, which often leads to high missed detection rates for smaller targets. In contrast, TBPAN (Figure 5d) effectively mitigates this issue, and also demonstrates superior performance in detecting the “ship” category as shown in the second row.

The role of SEAM in the backbone network is to promote representative feature learning and enhance the acquired semantic features, thereby improving the recognition performance of small targets. When the input size is 640, SEAM contributes an increase of 1.0% in overall Precision. For an input size of 1024, it contributes 0.1%, 0.2%, 0.2% and 0.2% to Precision, Recall, mA$P_{50}$ and mA$P_{50:95}$, respectively. As shown in Table 3, SEAM improves the for the “small vehicle” category by approximately 1.4%, demonstrating its effectiveness in recognizing small targets. Compared to the SE module at the same position, SEAM is lighter while maintaining similar accuracy. Finally, compared with the baseline model, the proposed method improves Precision, Recall, mA$P_{50}$ and mA$P_{50:9}$ by 0.5%, 1.0%, 0.5% and 0.6%, respectively, at an input size of 640. At the 1024 scale, RE-YOLO shows superior fault tolerance and feature-extraction capabilities, with improvements of 1.7%, 0.1%, 0.3% and 1.7% in Precision, Recall, mA$P_{50}$ and mA$P_{50:95}$, respectively.

In summary, each module of RE-YOLO has proved its effectiveness, and the overall network architecture achieves the best mAP score on the DOTA-v1.0 dataset, as well as the most stable Precision and Recall.

### 5.5. Comparing the Detection Performance of Different Models

In this section, a comprehensive comparison of the proposed RE-YOLO model with several existing models is presented. Theses models include YOLOv5 [22], LP-YOLO [3], YOLOv6 [61], YOLOv7 [21], YOLOv8 [23], YOLOv9 [70] and YOLOv10 [71]. To ensure a fair evaluation and minimize experimental variability, all models were trained under identical conditions using our specified training set. The best-performing configuration of each model was selected for comparison.

#### 5.5.1. Experiments on the DOTA-v1.0 Dataset

Table 3 and Table 4 present the detection results of RE-YOLO compared to various existing methods on the DOTA-v1.0 test set. When the input size is 640, RE-YOLO (without the SEAM module) achieves superior performance with a mA$P_{50}$ of 85.5%, mA$P_{50:95}$ of 63.6%, F1 score of 83.1% and a Recall of 79.9%. Compared to the baseline network YOLOv8s, RE-YOLO improves mA$P_{50}$ by 0.6%, mA$P_{50:95}$ by 0.8%, F1 score by 1.2% and Recall by 2.5%. In comparison with the suboptimal model YOLOv5s, YOLOv9s and YOLOv10s, RE-YOLO achieves improvements of 3.3%, 3.6% and 2.3% in mA$P_{50}$, as well as 7.8%, 6.9% and 5.5% in mA$P_{50:95}$, respectively. Notably, for the A$P_{50}$ metric, our method achieves the best results on relatively large objects such as “Large vehicle”, “Ship” and “Storage tank”, but shows a decrease for the “Small vehicle” category. This reduction can be attributed to the loss of local detail due to smaller feature maps at lower input scales. However, this issue can be significantly alleviated by incorporating the SEAM, which enhances A$P_{50}$ and A$P_{50:95}$ for “Small vehicle” by 2.8% and 1.4%, respectively.

At an input size of 1024, RE-YOLO achieves mA$P_{50}$, mA$P_{50:95}$, F1 score, Precision and Recall values of 86.9%, 65.8%, 84.0%, 88.1% and 80.3%, respectively. Compared to the baseline network YOLOv8s, our method improves mA$P_{50}$ by 0.3%, mA$P_{50:95}$ by 1.7% and F1 score by 0.8%. The substantial improvement in mA$P_{50:95}$ demonstrates that our method provides stronger performance at higher IoU thresholds, resulting in more accurate detection and greater robustness in complex scenes.

**Figure 6:**
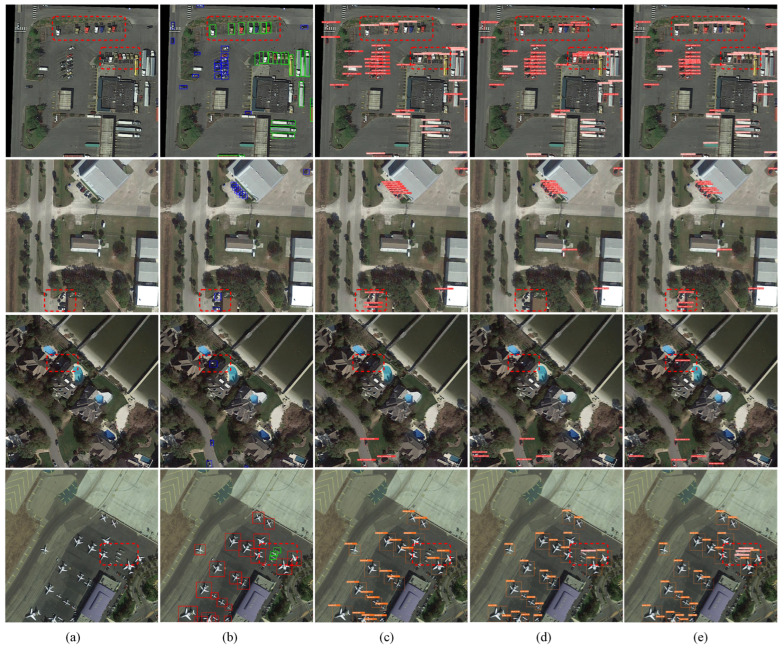
Comparing the detection results of different models on the DOTA-v1.0 dataset. (**a**) Source image. (**b**) Ground truth. (**c**) YOLOv5s. (**d**) YOLOv8s. (**e**) RE-YOLO.

In Figure 6, the detection results of the top three methods ranked by mAP are plotted, using samples with representative features from the test set of the DOTA-v1.0 dataset. The red box highlights areas of significant improvement, and the visual comparison aligns with the quantitative indicators. Observing the first row, it can be noted that the buildings in the remote sensing image are densely packed, and the target environment is relatively complex. The “small vehicle” and the large-sized “large vehicle” categories are often confused with medium-sized “large vehicles,” making it challenging to accurately distinguish between them. This scenario tests the network’s feature-extraction and -fusion capabilities. By leveraging TBPAN to emphasize low-level feature information and enhance the fusion with deep semantic information, the network gains a stronger ability to capture contextual information, enabling it to correctly distinguish nearly all medium-sized “large vehicles”.

The second and third rows illustrate cases where targets are obscured by shadows, making detection difficult. Our model better overcomes environmental challenges, demonstrating a higher prediction confidence score. In the fourth row, several white “large vehicles” are surrounded by “planes” of various shapes and sizes. Given their similar color and the smaller size of the “large vehicles” compared to the “planes”, these vehicles are often missed during detection. However, RE-YOLO (Figure 6e) shows strong adaptability and maintains high accuracy and precise localization in dense environments. Overall, RE-YOLO demonstrates superior accuracy compared to other networks in identifying and locating small and densely arranged targets in remote sensing images.

#### 5.5.2. Experiments on the SCERL Dataset

To verify the effectiveness and generalization ability of the proposed model on high-resolution remote sensing images, validation was conducted on the self-constructed SCERL dataset. The results, detailed in Table 5, reveal that while the performance differences among various networks have narrowed, our RE-YOLO model consistently outperforms all other methods across comprehensive metrics. Specifically, RE-YOLO achieves a mA$P_{50}$ of 45.8%, mA$P_{50:95}$ of 28.4%, Precision of 56.3% and Recall of 36.5%. Compared to the baseline model YOLOv8s, RE-YOLO improves mA$P_{50}$, mA$P_{50:95}$ and Precision by 1.0%, 1.5% and 4.9%, respectively, although there is a 2.8% decrease in Recall. It is worth noting that a relative decline in performance metrics was observed on the landslide dataset after the incorporation of the SEAM module. Moreover, while RE-YOLO achieves mA$P_{50}$ scores comparable to those of YOLOv6s and YOLOv7, it achieves these results with significantly fewer parameters and lower floating-point operations (FLOPs), highlighting its efficiency alongside its robust performance.

**Figure 7:**
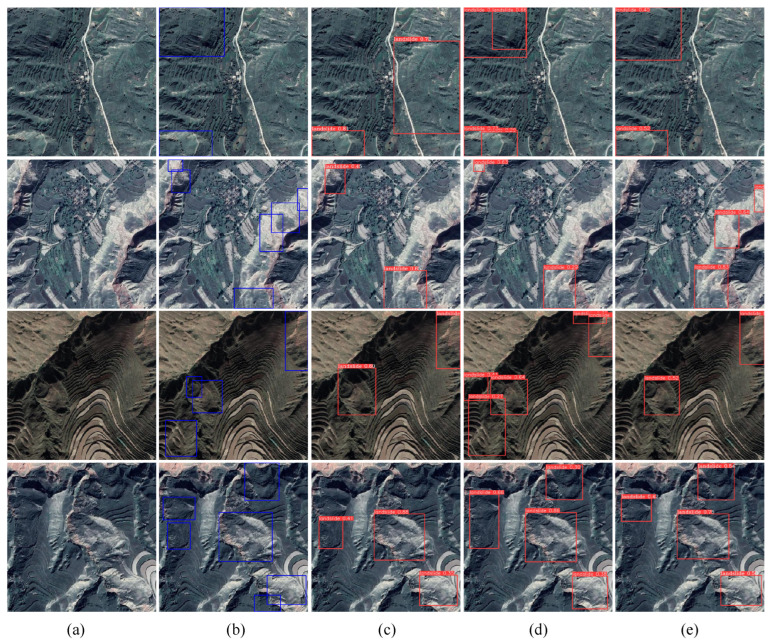
Comparing the detection results of different models on the SCERL dataset. (**a**) Source image. (**b**) Ground truth. (**c**) YOLOv6s. (**d**) YOLOv8s. (**e**) RE-YOLO.

Figure 7 illustrates the detection results of the top three mAP methods, using samples with representative features from the SCERL dataset. The first and second rows depict the detection performance of RE-YOLO and other methods on sparse landslide images, where our model clearly shows a higher confidence score, indicating greater certainty in the detected objects. The third to fifth rows demonstrate the detection performance on dense landslide images. Overall, RE-YOLO exhibits superior localization ability, accurately identifying dense and small-scale ground targets through its enhanced spatial and channel information-extraction capabilities.

### 5.6. Efficiency Analysis

To comprehensively compare the models, the number of parameters for the backbone network (Params-B), the total parameters for the model (Params-M) and the number of computations (FLOPs) were calculated for all networks under the same environment, using 640×640 resolution images as input. The results are presented in Table 6, where “t” (Tiny), “s” (Small) and “m” (Medium) represent different scales of backbone and neck networks. Ratio denotes the ratio of Params-B to Params-M.

For Params-B, our RE-YOLO achieves the lowest value among all models by using lightweight RE_CSP blocks and SEAM attention blocks as stacked modules in the backbone network. Regarding Params-M, YOLOv5s has the fewest parameters and computations due to its simple structure and the absence of a deep feature-extraction backbone and complex feature-fusion strategy. The ELAN module introduced in YOLOv7 enhances the model’s representation capacity by fusing gradient flow information across different layers but also significantly increases the number of parameters and computations. Our method maintains a moderate parameter count. Although the parameters and computations of the neck network are slightly increased due to a feature-fusion strategy tailored for remote sensing images, the overall parameter volume remains much lower than that of YOLOv6s and YOLOv7. In our model, the backbone network parameters constitute only 26.7% of the total parameters, which is significantly lower than the proportion in other models. Overall, our approach achieves the best detection performance with moderate Params and FLOPs.

## 6. Discussion and Conclusions

The proposed method effectively tackles the challenges inherent in remote sensing image detection, demonstrating strong generalization capabilities on the self-constructed SCERL dataset. Nevertheless, several notable limitations merit further discussion. Although our method achieves accurate detection in RSIs and outperforms other models, some redundancy persists in the neck network, resulting in a model size slightly larger than the baseline. This observation highlights significant opportunities for optimizing the overall architecture. Furthermore, the detection performance of our model reveals considerable potential for improvement across various dimensions, particularly concerning efficient network design and memory access costs. Moving forward, our future research will focus on model compression and lightweighting, and further refining our approach to enhance both accuracy and efficiency in remote sensing image-detection tasks.

This paper proposes a new framework for remote sensing image object detection, termed RE-YOLO. Initially, REM and RE_CSP blocks are designed to maintain the feature representation capacity while using moderate parameters. Moreover, RENet is constructed by stacking RE_CSP blocks as the backbone network to enable efficient feature extraction, and the SEAM is designed to capture long-range dependencies in spatial features, promoting more effective representative feature learning. In addition, a multi-scale feature-fusion strategy, TBPAN, is proposed specifically for remote sensing image detection. TBPAN enhances the extraction of shallow texture features and ensures the comprehensive fusion of multi-scale features. The effectiveness of each module is validated by extensive ablation experiments, demonstrating the superiority of the modules designed in this paper. Experimental results on two remote sensing image datasets with different resolutions, DOTA-v1.0 and SCERL, indicate that RE-YOLO achieves the highest detection accuracy in both mA$P_{50}$ and mA$P_{50:95}$. These results demonstrate the effectiveness of the RE-YOLO in RSOD, outperforming other models used in the experiments.

## Figures and Tables

**Table 1:** Description of abbreviations and symbols.

Abbreviation/Symbol	Full Name/Description
RSOD	Remote Sensing Object Detection
RSIs	Remote Sensing Images
CNNs	Convolutional Neural Networks
FPN	Feature Pyramid Network
⊙	Element-wise Multiplication
REM	Refined and Efficient Module
SEAM	Spatial Extracted Attention Module
TBPAN	Three-Branch Path Aggregation Network
IoU	Intersection Over Union
AP	Average Precision
mAP	Mean Average Precision
DWC	Depthwise Convolution
GC	Group Convolution
PWC	Pointwise Convolution
A$P_{50}$	Average Precision at an IoU threshold of 50%.
mA$P_{50}$	Mean Average Precision at an IoU threshold of 50%.
$R^{H\times W\times C}$	A 3D tensor with dimensions height, width and channel.
RE_CSP block	The block that consists of several REM and convolutional layers.

**Table 2:** Ablation experiment results for each component on DOTA-v1.0 dataset. ✓ and × indicate whether using the designed modules. “Replace” refers to applying a module with similar functionality compared to the designed modules from existing advanced methods to RE-YOLO. CSPDarkNet and PAFPN [23] serve as the backbone and neck networks of YOLOv8, respectively.

Method	Modules			Precision (%)	Recall (%)	F1 (%)	mA$P_{50}$ (%)	mA$P_{50:95}$ (%)	Params (MB)	FLOPs (G)
	RENet	TBPAN	SEAM							
Input Image Size: 640										
Baseline YOLOv8s				87.0	77.4	81.9	84.9	62.8	11.1	28.8
Replace	CSPDarkNet [23]	✓	×	87.1	79.4	83.1	85.2	63.5	13.9	40.0
	✓	PAFPN [23]	✓	85.9	77.9	81.7	84.8	61.6	9.1	22.6
	✓	BiFPN [27]	✓	86.1	77.8	81.7	85.0	62.4	5.5	21.0
	✓	✓	SE [52]	87.0	79.0	82.8	85.4	63.3	12.4	34.8
Remove	✓	✓	×	86.5	79.9	85.5	63.6	11.9	34.0	
RE-YOLO	✓	✓	×	86.5	79.9	83.1	85.5	63.6	11.9	34.0
RE-YOLO	✓	✓	✓	87.5	78.4	82.7	85.4	63.4	12.0	34.1
Input Image Size: 1024										
Baseline YOLOv8s				86.4	80.2	83.2	86.6	64.1	11.1	28.8
Replace	CSPDarkNet [23]	✓	×	87.3	80.8	83.9	87.1	65.4	13.9	40.0
	✓	PAFPN [23]	✓	87.9	80.0	83.7	86.5	64.6	9.1	22.6
	✓	BiFPN [27]	✓	87.2	80.3	83.6	86.5	65.3	5.5	21.0
	✓	✓	SE [52]	87.0	79.0	82.8	85.4	63.3	12.4	34.8
Remove	✓	✓	×	88.0	80.1	86.7	65.6	11.9	34.0	
RE-YOLO	✓	✓	×	88.0	80.1	83.8	86.7	65.6	11.9	34.0
RE-YOLO	✓	✓	✓	88.1	80.3	84.0	86.9	65.8	12.0	34.1

**Table 3:** Comparison with the single-model detectors on DOTA-v1.0 dataset. The best results are shown in bold, and the second best are underlined.

Method	Small Vehicle		Large Vehicle		Plane		Ship		Storage Tank		mA$P_{50}$ (%)	mA$P_{50:95}$ (%)
	A$P_{50}$	A$P_{50:95}$	A$P_{50}$	A$P_{50:95}$	A$P_{50}$	A$P_{50:95}$	A$P_{50}$	A$P_{50:95}$	A$P_{50}$	A$P_{50:95}$		
Image Size for Training: 640 × 640												
YOLOv5s	68.4	41.4	83.9	60.8	92.4	68.5	88.8	62.5	77.8	46	82.2	55.8
YOLOv5m	67.8	41.9	86.5	64.8	**93.8**	71.3	89.8	66.1	82.6	51.7	84.1	59.1
LP-YOLO	69.0	39.9	84.0	58.8	91.2	65.1	87.6	58.7	73.7	42.6	81.1	53.0
YOLOv6t	69.8	39.1	86.1	61.8	92.3	68.0	88.8	61.4	78.1	47.0	83.0	55.5
YOLOv6s	69.5	39.0	85.8	60.8	92.6	68.0	89.1	60.8	79.7	48.7	83.4	55.5
YOLOv7	68.2	40.8	86.2	63.9	93.2	69.3	89.2	64.6	77.3	45.5	82.8	56.8
YOLOv8s	74.2	47.6	86.5	68.0	92.2	72.0	91.2	68.8	81.0	**57.8**	84.9	62.8
YOLOv9s	70.6	42.0	84.0	62.3	91.4	68.2	89.4	64.3	74.2	46.5	81.9	56.7
YOLOv9m	71.3	43.2	86.6	65.2	93.2	70.5	90.5	66.4	78.5	48.7	84.0	58.8
YOLOv10s	70.2	43.1	**87.6**	66.3	92.1	68.0	89.5	65.1	76.8	48.2	83.2	58.1
RE-YOLO	71.7	47.0	87.2	**69.3**	93.0	**74.2**	**91.7**	**70.4**	**83.9**	57.2	**85.5**	**63.6**
RE-YOLO + SEAM	**74.5**	**48.4**	86.6	68.6	92.8	73.3	91.3	69.9	81.7	56.8	85.4	63.4
Image Size for Training: 1024 × 1024												
YOLOv5s	70.3	43.3	86.0	65.2	94.3	69.9	90.1	66.5	82.1	53.3	84.6	59.6
LP-YOLO	74.9	43.4	86.0	60.7	90.8	63.0	88.7	61.6	77.5	46.7	83.6	55.1
YOLOv8s	**76.6**	49.5	87.0	69.6	92.6	73.2	91.8	71.1	**84.7**	57.0	86.6	64.1
YOLOv9m	75.3	47.3	86.5	66.0	92.8	70.6	90.9	68.2	82.1	53.6	85.5	61.2
YOLOv10s	76.4	47.2	85.6	64.7	91.9	68.2	89.3	65.4	79.9	50.3	84.6	59.2
RE-YOLO	76.0	49.8	87.7	70.8	**93.9**	**75.5**	**92.4**	**72.6**	83.7	59.5	86.7	65.6
RE-YOLO + SEAM	76.1	**50.2**	**88.5**	**71.2**	93.6	75.4	92.2	72.5	83.7	**59.8**	**86.9**	**65.8**

**Table 4:** Comparison with the single-model detectors on DOTA-v1.0 dataset. Pre, Rec and F1 denote Precision, Recall and F1 score, respectively. The best results are shown in bold, and the second best are underlined.

Method	Small Vehicle		Large Vehicle		Plane		Ship		Storage Tank		Pre (%)	Rec (%)	F1 (%)
	Pre	Rec	Pre	Rec	Pre	Rec	Pre	Rec	Pre	Rec			
Image Size for Training: 640 × 640													
YOLOv5s	73.6	65.9	87.6	79.7	94.3	87.8	92.8	84.7	92.3	66.6	**88.1**	77.0	82.2
LP-YOLO	73.2	65.7	85.3	78.4	93.2	85.5	91.7	82.8	93.0	60.2	87.3	74.5	80.4
YOLOv6t	72.0	65.4	84.7	80.7	95.0	88.0	92.3	85.2	83.9	69.9	85.6	77.8	81.5
YOLOv6s	71.4	67.0	83.0	81.6	93.3	88.6	91.5	86.1	88.5	69.7	85.5	78.6	81.9
YOLOv7	64.2	67.4	83.8	81.4	94.2	88.0	92.3	86.0	92.3	64.8	85.4	77.5	81.2
YOLOv8s	70.2	72.7	85.3	80.7	93.7	86.0	92.4	85.2	93.4	62.5	87.0	77.4	81.9
YOLOv9s	69.6	70.8	84.6	80.0	93.1	86.4	92.5	85.0	95.0	61.1	87.0	76.7	81.5
YOLOv9m	66.6	73.1	82.7	83.5	95.0	87.6	92.4	86.5	93.3	64.5	86.0	79.0	82.3
YOLOv10s	65.1	72.5	86.6	82.2	92.8	86.6	92.5	85.2	88.6	64.5	85.1	78.2	81.5
RE-YOLO	69.5	73.1	85.4	83.0	94.2	87.9	91.7	86.5	91.9	69.1	86.5	**79.9**	**83.1**
RE-YOLO + SEAM	72.0	72.7	86.1	80.9	94.2	87.1	92.3	85.8	92.8	65.5	87.5	78.4	82.7
Image Size for Training: 1024 × 1024													
YOLOv5s	70.2	70.8	88.6	80.5	94.8	87.6	93.4	86.5	95.8	70.0	**88.5**	79.1	83.5
LP-YOLO	74.9	69.4	85.2	80.5	94.3	83.1	92.4	83.7	94.0	64.1	88.2	76.2	81.8
YOLOv8s	69.5	74.6	84.9	82.2	93.1	87.1	91.6	86.8	92.7	70.0	86.4	80.2	83.2
YOLOv9m	70.4	73.6	84.9	82.2	93.9	87.4	92.6	86.8	93.1	68.1	87.0	79.6	83.1
YOLOv10s	74.8	74.4	84.2	79.2	91.6	85.1	90.4	85.3	87.74	67.1	85.8	78.2	81.8
RE-YOLO	73.4	74.2	86.2	82.8	94.5	88.5	92.6	87.4	93.5	67.8	88.0	80.1	83.9
RE-YOLO + SEAM	72.7	74.2	86.4	84.0	94.3	88.1	92.7	87.0	94.2	68.1	88.1	**80.3**	**84.0**

**Table 5:** Comparison with the single-model detectors on SCERL dataset.

Method	mA$P_{50}$ (%)	mA$P_{50:95}$ (%)	Precision (%)	Recall (%)	Params (MB)	FLOPs (G)
YOLOv5s	42.6	22.4	48.9	46.0	7.0	15.8
YOLOv6s	45.8	24.7	46.6	52.5	18.8	48.9
YOLOv7	45.7	24.9	48.7	49.2	39.9	109.8
YOLOv8s	44.8	26.9	51.4	39.3	11.1	28.8
YOLOv9s	41.3	22.8	44.4	45.7	6.2	22.1
YOLOv9m	43.3	22.4	50.2	46.6	16.5	60.0
YOLOv10s	40.6	20.8	45.1	45.2	8.1	24.4
RE-YOLO	45.8	28.4	56.3	36.5	11.9	34.0
RE-YOLO + SEAM	44.2	27.5	53.9	37.2	12.0	34.1

**Table 6:** Efficiency analysis.

Method	Backbone	Params-B (MB)	Params-M (MB)	Ratio (%)	FLOPs (G)
YOLOv5s	CSPDarkNet-s-C3	4.0	7.2	55.5	15.9
YOLOv5m	CSPDarkNet-m-C3	12.2	21.2	57.5	49.2
YOLOv6t	EfficientRep-t	6.6	10.6	62.2	27.64
YOLOv6s	EfficientRep-s	12.3	18.8	65.1	48.9
YOLOv7	E-ELAN	20.9	39.9	52.3	109.8
YOLOv8s	CSPDarkNet-s-C2F	5.1	11.1	45.9	28.8
RE-YOLO	RENet	3.1	11.9	26.1	34.0
RE-YOLO + SEAM	RENet	3.2	12.0	26.7	34.1

## Data Availability

Code is available at https://captain-whu.github.io/DOTA/dataset.html and https://github.com/Moore-K2/RE-YOLO accessed on 27 October 2024.
